# Design, Methods, and Select Baseline Results from a School Nutrition Project for Adolescents in Bangladesh

**DOI:** 10.1016/j.cdnut.2023.100070

**Published:** 2023-03-30

**Authors:** Maku E. Demuyakor, Chowdhury Jalal, Anne M. Williams, Kimberley P. Bouckaert, Ralph D. Whitehead, Muhammad M. Bhuiyan, Saiqa Siraj, Riffat Ara, Vanessa Pike, Maria Elena D. Jefferds

**Affiliations:** 1 Nutrition Branch, Centers for Disease Control and Prevention, Atlanta, GA, United States; 2 McKing Consulting Corporation, Atlanta, GA, United States; 3 Nutrition International, Ottawa, Ontario, Canada; 4 Nutrition International, Gulshan 1, Dhaka, Bangladesh; 5 Centre for Qualitative Research, Ibrahimpur, Dhaka, Bangladesh; 6 BRAC Health, Nutrition and Population Program, Mohakhali BRAC Center, Dhaka, Bangladesh

**Keywords:** Bangladesh, adolescent, anemia, micronutrient status, school nutrition intervention, water, sanitation, hygiene

## Abstract

**Background:**

The School Nutrition for Adolescents Project (SNAP) provided weekly iron and folic acid (WIFA) supplementation and menstrual hygiene management (MHM) support for girls; actions to improve water, sanitation, and hygiene (WASH) practices; and behavior change interventions to adolescents aged 10–19 y in 65 intervention schools in 2 districts of Bangladesh.

**Objectives:**

We aimed to describe the project design and select baseline results of students and school project implementers.

**Methods:**

Girls (n = 2244) and boys (n = 773) in 74 schools (clusters) and project implementers [headteachers (n = 74), teachers (n = 96), and student leaders (n = 91)] participated in a survey assessing nutrition, MHM, and WASH knowledge and experience. Hemoglobin, inflammation-adjusted ferritin, retinol-binding protein, and serum and RBC folate (RBCF) levels in girls were measured. School WASH infrastructure was observed and drinking water was tested for *E. coli*.

**Results:**

IFA and deworming tablet intake in the last 1 and 6 mo were 4% and 81% for girls and 1% and 86%, respectively. Applying the Minimum Dietary Diversity for Women (MDD-W) tool, most (63%–68%) girls and boys achieved minimum dietary diversity. Fewer adolescents (14%–52%) had ever heard of anemia, IFA tablets, or worm infestation than project implementers (47%–100%). Girls (35%) missed school during menstruation; 39% reported of ever leaving school due to unexpected menstruation. The micronutrient status and deficiency severity varied: anemia (25%), RBCF insufficiency (76%), risk of serum folate deficiency (10%), deficiencies of iron (9%), and vitamin A (3%). WASH in school sustainable development goal (SDG) indicators achievement varied: basic drinking water service (70%), basic sanitation service (42%), and basic hygiene service (3%); 59% of sampled drinking water access points complied with WHO *E. coli* standards.

**Conclusions:**

There is room for improvement of nutrition and health awareness, practices, micronutrient status, SDG basic WASH in-school services, and *E coli* contamination in school drinking water.

This trial was registered in clinicaltrials.gov as NCT05455073.

## Introduction

In Bangladesh, anemia remains a public health problem and affects about 26% of nonpregnant nonlactating (NPNL) women, including adolescent girls [1]. Anemia is a cause for concern for adolescent girls due to the additional requirements for iron to replace blood loss during menstruation to compensate for inadequate dietary quality and intake and also because it is a time of rapid growth [2,3]. Furthermore, the occurrence of pregnancy during adolescence poses additional risks of iron deficiency (ID), leading to an increased risk of maternal and infant morbidity and mortality, including low birthweight and preterm babies [[4], [5], [6]], which can contribute to the intergenerational cycle of malnutrition. Although the causes of anemia are multifaceted, approximately 25%–50% of anemia is caused by iron deficiency globally among young children and women aged 15–49 y [7]; however, this may not be the case in Bangladesh as elevated iron levels are present in some groundwater supplies from tube wells nationwide [8]. In low- and middle-income countries (LMICs), including Bangladesh, additional factors contributing to IDA among adolescent girls include soil-transmitted helminths infections; genetic hemoglobin blood disorders; suboptimal personal agency and empowerment; and poor understanding and practices of good nutrition, sanitation, and hygiene [3,9]. Inadequate amounts of folate in the diet or poor folate status may also cause anemia and contribute to neural tube defects during pregnancy and other negative birth outcomes [10].

Addressing the nutrition and related needs of adolescents could be an important initiative for increasing national productivity and for breaking the cycle of intergenerational malnutrition, chronic diseases, and poverty [11,12]. To respond to these diverse needs of adolescents including anemia reduction, the Government of Bangladesh (GoB) in 2011 instituted a national policy for weekly iron and folic acid (WIFA) supplementation program for both in-school and out-of-school adolescent girls aged 10–19 y [13]; both IFA and MMS supplementation are also key strategies in the National Strategy for Adolescent Health 2017–2030 [14]. To demonstrate the feasibility of this initiative and to provide recommendations to the GoB about how best to implement its adolescent WIFA policy through scale-up of an integrated program, Nutrition International (NI) in collaboration with BRAC established a demonstration project called the School Nutrition for Adolescents Project (SNAP) in 2019. SNAP combined a nutrition-specific and nutrition-sensitive intervention model in 90 schools (65 intervention-receiving schools) to deliver an integrated package for adolescent girls and boys including free WIFA supplementation to girls and menstrual hygiene management (MHM) support for girls to improve school attendance during menstruation; support to improve water, sanitation, and hygiene (WASH) practices; and behavior change interventions (BCI) for dietary change of adolescents as part of a research trial. Evaluation of both the outcome and process of the first year (2019–2020) of the project implementation was planned by NI in collaboration with the Bangladesh government’s National Nutrition Services (NNS), Institute of Public Health Nutrition (IPHN), Ministry of Health and Family Welfare, and the Directorate of Secondary and Higher Education, Ministry of Education (DSHE), with technical assistance from the U.S. CDC and the CDC Foundation.

The SNAP outcome evaluation is registered at https://clinicaltrials.gov as NCT05455073. There were 2 types of intervention packages: 1) a full package (FP) intervention that included all components of the project (WIFA supplementation, MHM, WASH, and BCI), and 2) a limited package (LP) intervention that included only WIFA supplementation and BCI components. The secondary audiences were social influencers of the adolescents, including the school head teachers, teachers and student leaders, parents, community leaders, and policymakers who were sensitized through training and social mobilization sessions around IFA supplementation, MHM, WASH, and nutrition education.

The project implementing partner was BRAC (Health, Nutrition, and Population Program), the largest nongovernmental organization in the world based in Bangladesh, that rolled out the interventions across 50 schools in the Joypurhat district and 15 schools in the Sirajganj district, both in the Rajshahi Division of Bangladesh.

The SNAP project was implemented on a rolling basis in the selected FP and LP schools from October 2019 and was intended to roll out for a full year; however, all project activities, including WIFA distribution to girls and BCI classroom sessions to girls and boys, were abruptly halted in March 2020 because of school closures caused by the COVID 19 pandemic, and the SNAP project and trial did not re-start.

The objectives of this manuscript are to describe the outcome evaluation study design, methods, and select descriptive results from the baseline survey. Although the intervention was stopped prematurely, it is anticipated that findings from the baseline survey will add to the literature on adolescents’ and school intervention implementers’ knowledge, attitude, and practices (KAP) of nutrition and health, adolescent girls’ micronutrient status in rural Bangladesh, and the WASH environment of schools, which will be useful for designing and implementing effective integrated nutrition and health programs and policies to address the burden of malnutrition among adolescents in this and similar populations.

## Methods

### Study site and population

Although the SNAP project was implemented in both Joypurhat and Sirajgonj districts, Joypurhat was selected as the evaluation intervention district and is the focus of this paper. Joypurhat is a relatively small district in the Rajshahi division, North-East region of Bangladesh. The inhabitants are mainly farmers, with the majority farming a variety of crops including rice, potatoes, and sugarcane [15].

### Study design

The design of the outcome evaluation survey was a cross-sectional pre- and post-survey cluster randomized trial with 3 equal-size study arms: a FP intervention, a LP intervention, and a control (C) group that did not receive any intervention component. The population for the outcome evaluation included adolescents in grades 8 and 9 (majority aged 14–19 y) in 75 schools in Joypurhat (25 schools in each arm). Head teachers, assigned intervention teachers, and assigned intervention student leaders [2 teachers and 2 student leaders per relevant content area for nutrition counseling and WIFA distribution, MHM (female only), and WASH] in intervention schools also participated in the evaluation.

### Study participants’ inclusion and exclusion criteria

Only adolescents enrolled in grades 8 or 9 of the 2019–2020 academic year were included in the baseline survey. To be eligible for participation, each adolescent had to have been randomly selected, been present on the day(s) of the survey, given verbal assent, and have parental/guardian written consent. At the school level, the head teacher, all intervention-assigned teachers, and student leaders in the various grades were eligible to participate in the surveys if they had verbal informed consent or parental written consent and verbal assent. Adolescents who enrolled after random selection of participants were not eligible.

### Sample size and power

Schools were considered as clusters for sampling purposes. Sample size calculations were powered to detect differences in mean anemia prevalence among adolescent girls between the FP and the control schools. The schools receiving the LP intervention were not considered in sample size estimates as they were not planned to be included in the main statistical analysis, which assumed comparison between FP and control clusters only. The study was powered to detect a 7% point change in anemia prevalence between the 2 groups. The intracluster correlation was estimated to be 5% with a cluster size of 36 and a design effect of 2.75. Assuming 26% baseline prevalence [1] with 80% power and a 2-sided 95% significance level, 50 clusters (schools; 25 per intervention arm) were required for the 2-group comparison. Based on the equation [16],

$$m=\frac{{\left(z_{1-\frac{\alpha }{2}}+z_{1-\beta }\right)}^{2}\times \left[p_{0}\left(1-p_{0}\right)+p_{1}\left(1-p_{1}\right)\right]\times DEFF}{n{\left(p_{1}-p_{0}\right)}^{2}}$$

where m is the number of clusters required per arm; $p_{0}$ = the prevalence of anemia among unexposed (control); $p_{1}$ is the prevalence of anemia among exposed (treatment group); and DEFF = 1 + σ×(n−1), where σ is the intracluster correlation and n is the cluster size.

Given that there were 3 arms of the study, 75 schools were randomized to either the full package, limited package, or control groups to have a balanced (25 clusters/group) design. Additionally, a random sample of 12 adolescent boys per school (6 per grades 8 and 9) were selected across the 75 schools for KAP of nutrition and health topics and interventions as they were included in WASH, BCI, and KAP interventions within schools. We aimed to achieve a sample size of 150 boys in 8th and 9th grades per intervention arm (300 total per arm).

### Sampling procedures

A 2-stage sampling strategy was followed. First, all 75 schools in 4 out of 5 upazilas in Joypurhat were randomized into the 3 intervention arms using the RAND function in Microsoft Excel (Microsoft Corporation). To allow consistency across the upazilas, the largest and most urban upazila, Joypurhat Sadar, was excluded. Second, within the randomized schools, adolescents were selected to participate by simple random sampling using the SAS software version 9.4 (SAS Institute) [17] based on sex and grade. A maximum of 48 eligible adolescents were selected per school from grades 8 and 9; 18 girls and 6 boys from each grade. In schools where there were ≤18 girls or 6 boys with parental consent, all girls or boys were selected. On the day of the survey, with the help of the assigned teachers, all selected adolescents with parental consent who were present in school were invited to participate. Absent adolescents were not replaced on the list but were recontacted to participate during a mop-up survey after the 1st round of data collection. Additionally, for schools where fewer than 18 girls or 6 boys per grade had parental consent during the 1st round of data collection, reminders were sent to parents or guardians to return consent forms, and the selection process was repeated during mop-up to achieve the required sample sizes.

### Field teams and training

For the survey, there was a total of 5 field teams with 6 enumerators each to interview girl and boy students, 1 enumerator to carry out teacher and student leader interviews and observations of the school environment, 2 laboratory technicians, and 1 team lead. All interviewers had prior interviewing experience and training. Each field team worked in 1 school and collected all data electronically the same day into android-enabled devices using an Open Data Kit [18]. Teams were trained on ethical considerations and the consent process, inclusion and exclusion criteria, interviewing techniques, and role-playing. The training included classroom-based training, field-based practical training, and pilot testing. Laboratory technicians were trained on the collection and processing of venous blood specimens, universal precautions, use of the HemoCue Hb-301 photometer, cold chain procedures, and water collection and testing techniques. All aspects of the fieldwork were pilot-tested during the training week with students and teachers in 2 local schools near the training center in Dhaka.

### Survey instruments

At baseline, several questionnaires were administered at the school to adolescents (sex-specific), head teachers, assigned teachers, and student leaders. Observations of preintervention toilet and hand washing facilities and BCI materials were made in each school. All questionnaires were developed in Microsoft Word in English and translated into Bangla and back-translated by an independent source. Enumerators administered questions orally in Bangla.

To assess the quality of groundwater used by the household, adolescents were asked about their perception of the level of iron in the tube well or dug well used by the household on a 4-point scale (“none”, “a little”, “a medium amount”, and “a lot”). This was based on the question “How much iron do you think is in the water that you pump from this drinking water tube well/dug well?”, which was validated among adult women in rural Bangladesh against a field-based calorimetric test kit in a study by Merrill et al. [19].

Dietary intake was assessed by a modified food frequency questionnaire (FFQ) including iron-rich foods [20] in a 24-h period. The FFQ was developed and adapted into the Bangladeshi context by the survey team; however, it was not validated. Adolescents were asked to recall various food groups and beverages, including animal source foods, dark leafy greens, nutrient-poor foods (sugar sweetened beverages, noodles, chips, and biscuits), and pica (intake of soil, clay, uncooked rice, starch, or ice).

Project implementers (head teachers, assigned teachers, and student leaders) were administered questionnaires similar to those of adolescents, and asked about pre-existing activities that potentially could overlap with the various planned SNAP intervention areas.

### Water testing and biological data collection

Trained enumerators collected samples from up to 2 student drinking water stations per school. Drinking water quality was assessed using the Aquagenx portable compartment bag test kit [21]. Schools were informed of the results of the water testing. To raise awareness on the quality of drinking water, each school was provided evidence-based general guidelines on point-of-use water storage and treatment to prevent and/or treat *E. coli.*

Following standard procedures, the field laboratory technician collected 6 mL of venous blood specimen for girls using the intravenipuncture technique. Butterfly needles and 2 vacutainers (one 3-mL potassium ethanediaminetetraacetic acid (K_2_EDTA) coated tube and one 3-mL silicone-coated tube) were used for the blood collection. A few drops of the collected sample in the K_2_EDTA vacutainers were used on the spot to determine hemoglobin concentration, whereas the remaining samples were labeled and stored inside a cold box with a thermometer to monitor temperature for later processing in the field office by the lab coordinator. The K_2_EDTA vacutainers were used to prepare a whole blood lysate for the analysis of RBC folate. This was prepared before centrifugation at the end of day. The silicone-coated vacutainer was centrifuged at the end of the day to obtain serum for laboratory analysis.

Whole blood was assessed for hemoglobin (Hb) using the HemoCue Hb-301 photometer (HemoCue Ltd.). Biomarkers of iron including ferritin and sTfR and vitamin A (RBP) and indicators of inflammation [CRP and alpha 1-acid glycoprotein (AGP)] were assessed using a sandwich ELISA method [22]. The folate status was assessed using a microbiological assay to test for RBC folate and serum folate [23].

### Ethical considerations

The study protocol for the outcome evaluation was approved by the Bangladesh Medical Research Council’s Institutional Review Board, Dhaka, Bangladesh. Written informed consent was obtained from the school by the head teacher for the school’s participation and from parents/guardians of all adolescents in 8th and 9th grade and student leaders. All selected adolescents and student leaders provided informed verbal assent, and assigned intervention teachers gave informed verbal consent. The consent forms were written in the local language (Bangla) in a format that could be easily understood by study participants with little or no education. The data were uploaded to a secure server and were inaccessible to field teams and supervisors. A password-protected database was only accessible to select members of the evaluation team.

### Data analysis

Descriptive statistics were analyzed in SAS (version 9.4). All reported proportions accounted for clustering by estimating the variance among each cluster (school) and excluding missing values for any cluster variable during variance estimation. Reported counts [sample sizes (*n*) in all tables] are unweighted.

A minimum dietary diversity score was derived from food groups consumed in the previous 24 h. The food groups were adapted from the Minimum Dietary Diversity for Women (MDD-W) tool, validated for women of reproductive age (15–49 y) [24]. Rich sources of heme iron included red meats and organ meats. Fair sources of heme iron included white meats, poultry, fish, and eggs. Sources of nonheme iron included dark green leafy vegetables and legumes. Pica practice is described as the intake of soil, clay, uncooked rice, starch, or ice.

WASH standards and criteria were adapted from WHO/UNICEF [25].

Anemia was defined using age-specific cutoffs for hemoglobin concentration – adolescent girls aged 10–11 y: <11.5 g/dL; adolescent girls aged 12–19 y: <12.0 g/dL. Mild anemia in girls aged 10–11 y: Hb = 11.0-11.4 g/dL; that in girls aged ≥12 y: Hb = 11.0–11.9 g/dL. Moderate anemia is defined as a Hb concentration of 8.0–10.9 g/dL. Severe anemia is defined as a Hb concentration of <8.0 g/dL [26]. No adjustment for altitude was warranted for the Joypurhat district since elevation was not above 1000 m. Smoking among adolescents is rare in Bangladesh and was not assessed or accounted for in the anemia calculations. ID was defined using inflammation-adjusted [[27], [28], [29]] ferritin of <15 μg/L [30] and sTfR of >8.3 mg/L [22]. The RBC folate concentration cutoff for folate insufficiency in RBC for preventing neural tube defects was <748 nmol/L (5MeTHF assay-specific cutoff) [31]. The risk of serum folate deficiency was defined as <7 nmol/L (deficiency based on hematologic indicator) [23]. The serum retinol cutoff of <0.7μmol/L to define vitamin A deficiency was used to define vitamin A deficiency using RBP [32]. CRP and alpha 1-acid glycoprotein (AGP) were used to adjust serum ferritin and sTfR [29].

There were 741 samples (34.2%) with CRP values below the lower limit of detection (LLOD). To standardize the data and avoid bias, a random number single imputation ranging from 0 to 0.10 was used to replace those CRP values less than the LLOD [33] for subsequent analyses. There were 12 samples (0.6%) with sTfR and 2 (0.1%) with RBP values outside the upper limits of 40.0 mg/L and 3.5 umol/L, respectively. These values were substituted with 40.1 and 3.51, respectively, and included in the analysis to preserve the original sample size.

## Results

### Response rates and participant characteristics

We describe here select descriptive results from the baseline survey combined for populations (not by trial design as it was stopped) due to the limited information on adolescents regarding the intervention topics. However, Supplemental Tables 1 and 2 show the sex and age of participants by the intervention arm.

At baseline, 74 out of the 75 schools selected agreed to participate in the survey (1 LP school declined). A total of 12,691 adolescents were eligible to participate from grades 8 and 9 in all 74 schools, and 71.2% had parental consent to participate. Among the eligible adolescents, a total of 18 girls and 6 boys per grade were randomly selected to participate in the survey. In about 50% of the schools, there were fewer than 18 girls or 6 boys enrolled per grade. When this occurred, all eligible girls and boys in these grades were invited to participate. Thus, 3717 eligible participants were invited to participate, and the response rates for a completed questionnaire were 85% for girls and 73% for boys (Supplemental Figure 1).

About 96% of the participating girls gave a biological specimen at baseline. The total number of samples used for analysis after removing missing or mislabeled data were 2150 for hemoglobin, 2038 for iron, and 2159 for folate.

Among project implementers (Supplemental Figure 2), 74 head teachers, 46 nutrition-WIFA teachers, 25 MHM teachers, and 25 WASH teachers agreed to participate in the survey. No additional implementer interviews occurred in control schools. For brevity, all teacher categories have been combined in this paper (*N* = 96). The number of student leaders who participated was 91, with 66 out of 75 from the FP schools and 25 out of 25 from the LP schools.

### Exposure to nutrition and worm infestation

Table 1 shows the mean age, sex, and exposure to various health and nutrition topics among adolescents and implementers at baseline. A little over half of the student leaders and teachers were female compared to only 7% of headteachers. The mean age of girls, boys, and student leaders was about 14 y, whereas that of head teachers and teachers ranged from 41 y to 49 y.

**TABLE 1:** Sex, age and exposure to various health and nutrition topics among adolescents and implementers at baseline for the School Nutrition for Adolescents Project (SNAP) in Joypurhat, Bangladesh, 2019

Project domains	Adolescent girls N = 2244	Adolescent boys N = 773	Student leaders N = 91	Headteachers N = 74	All teachers (Nutrition-WIFA, WASH, MHM) N = 96
Female sex, n (%)	100.0	0.0	59.3	6.8	55.2
Mean age, y	14.1	14.3	14.2	49.3	41.4
Ever heard of anemia, n (%)	743 (33.1)	209 (27.0)	66 (72.5)	63 (85.1)	86 (89.6)
Ever heard of iron folic acid tablet, n (%)	700 (31.2)	110 (14.2)	43 (47.3)	69 (93.2)	87 (90.6)
Ever heard of deworming tablet, n (%)	1896 (84.5)	658 (85.1)	90 (98.9)	74 (100.0)	96 (100.0)
Ever talked to someone about menstruation, n (%)	1992 (88.8)	198 (25.6)	61 (67.0)	Not asked	Not asked
Ever heard of worm infestation, n (%)	1105 (49.2)	400 (51.7)	65 (71.4)	56 (75.7)	75 (78.1)
Ever had worm infestation^1^, n (%)	660 (59.7)	282 (70.5)	35 (53.8)	32 (57.1)	52 (69.3)

MHM, menstrual hygiene management; WASH, water, sanitation and hygiene.

1 Among those who had heard of worm infestations.

Generally, adolescents compared with student leaders and teachers reported lower levels of ever hearing of anemia, IFA tablets, or worm infestation. Nine out of 10 girls had ever talked to someone about menstruation in contrast to only about a quarter of boys. Among those who had heard of worm infestations, the majority of girls and boys, student leaders, and teachers never had a worm infestation (54%–71%).

When asked about their IFA tablet use in the previous month or deworming tablet intake in the last 6 mo, 4% and 1% of girls and boys reported intake of IFA tablets, respectively, and 81% and 86% of girls and boys reported intake of deworming tablets, respectively (data not shown).

**FIGURE 1:**
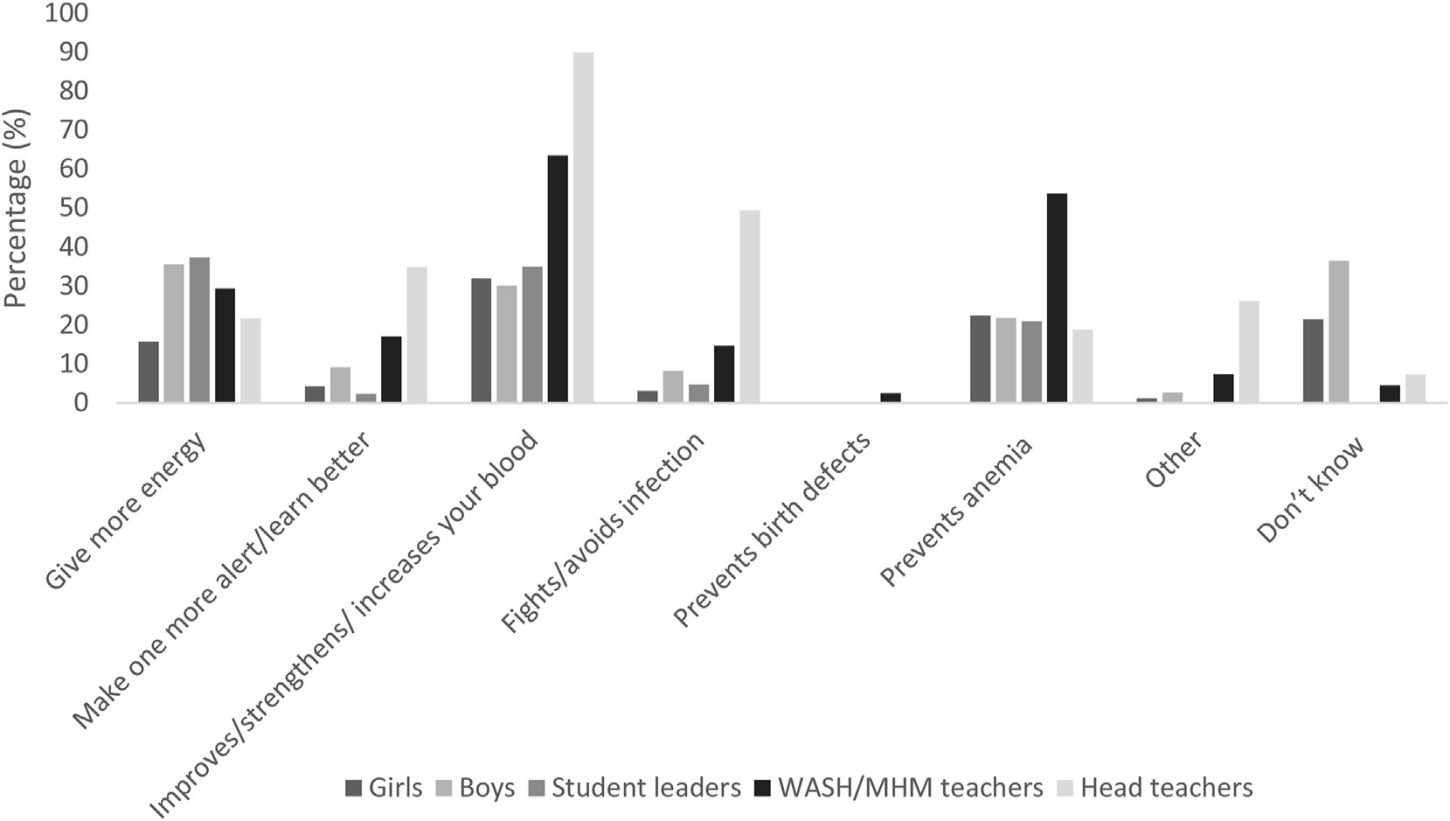
Reported benefits of iron and folic acid (IFA) tablet consumption among adolescents and implementers who had heard of IFA tablets at baseline for the School Nutrition for Adolescents Project (SNAP), Joyphurat district, Bangladesh, 2019. Girls: *N* = 2244; boys: *N* = 773; student leaders: *N* = 91; WASH/MHM teachers: *N* = 50; head teachers: *N* = 74. MHM, menstrual hygiene management; WASH, water, sanitation, and hygiene.

Figure 1 shows the reported benefits of IFA tablet consumption among those who had heard of IFA. Around 1 out of 3 of all adolescents reported that IFA improves, strengthens, or increases blood, whereas this was reported by >2 out of 3 of teachers. Around 1 in 5 adolescents and head teachers stated that IFA prevents anemia, as did around 1 in 2 WASH/MHM teachers (data not available for nutrition-WIFA teachers). Among boys, student leaders, and WASH/MHM teachers, 29%–37% reported that IFA gives 1 more energy and 35% of head teachers reported that it makes one more alert or one can learn better.

### Perceived iron content and characteristics of household drinking water among adolescent girls and boys

Among adolescent girls and boys who used a tube well as the main source of household drinking water (*N* = 1653 and 630, respectively), about a fifth reported that they thought there was iron in the water, while <10% thought that the drinking water tasted or smelled like rust or had a red or rusty color (data not shown).

### Reported intake of select food groups in the prior 24 h among adolescent girls and boys

**FIGURE 2:**
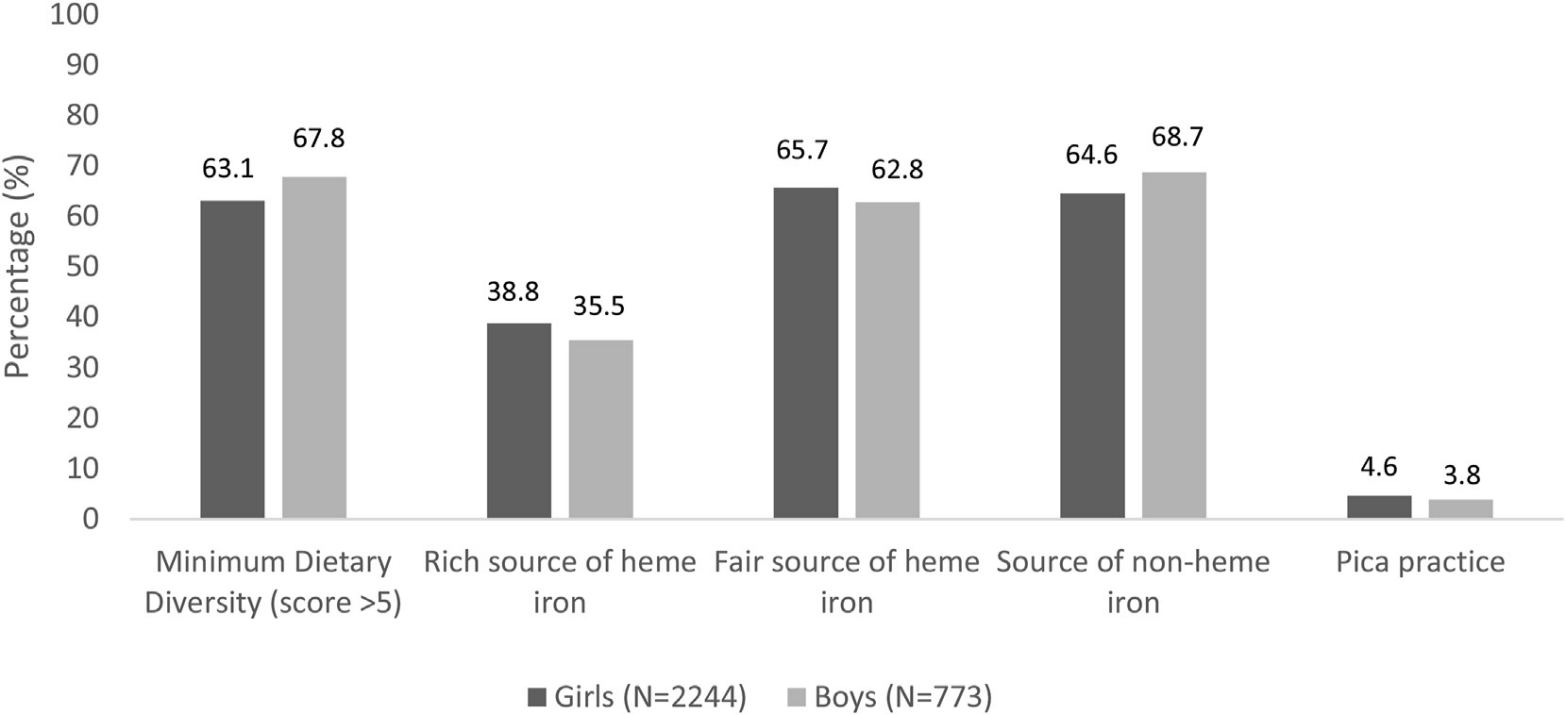
Reported intake of select food groups in the prior 24 h among adolescent girls and boys at baseline for the School Nutrition for Adolescents Project (SNAP), Joyphurat district, Bangladesh, 2019. The dietary diversity score is based on 10-food groups with categories designated by the Minimum Dietary Diversity for Women (MDD-W) tool, and a score greater than 5 is considered adequate [24]. Rich sources of heme iron included red meats and organ meats. Fair sources of heme iron included white meats, poultry, fish, and eggs. Sources of nonheme iron included dark green leafy vegetables and legumes. Pica practice is described as the intake of soil, clay, uncooked rice, starch, or ice.

On average, adolescent girls and boys consumed 5.2 and 5.3 out of the 10 food groups, respectively, in the previous 24 h before the survey. Over 60% of both girls and boys had a minimum dietary diversity score of >5 food groups, and about the same proportion consumed fair sources of heme iron and nonheme iron. Around 36% to 39% of boys and girls consumed rich sources of heme iron, whereas pica practice was uncommon (Figure 2).

### Exposure to WASH practices

**FIGURE 3:**
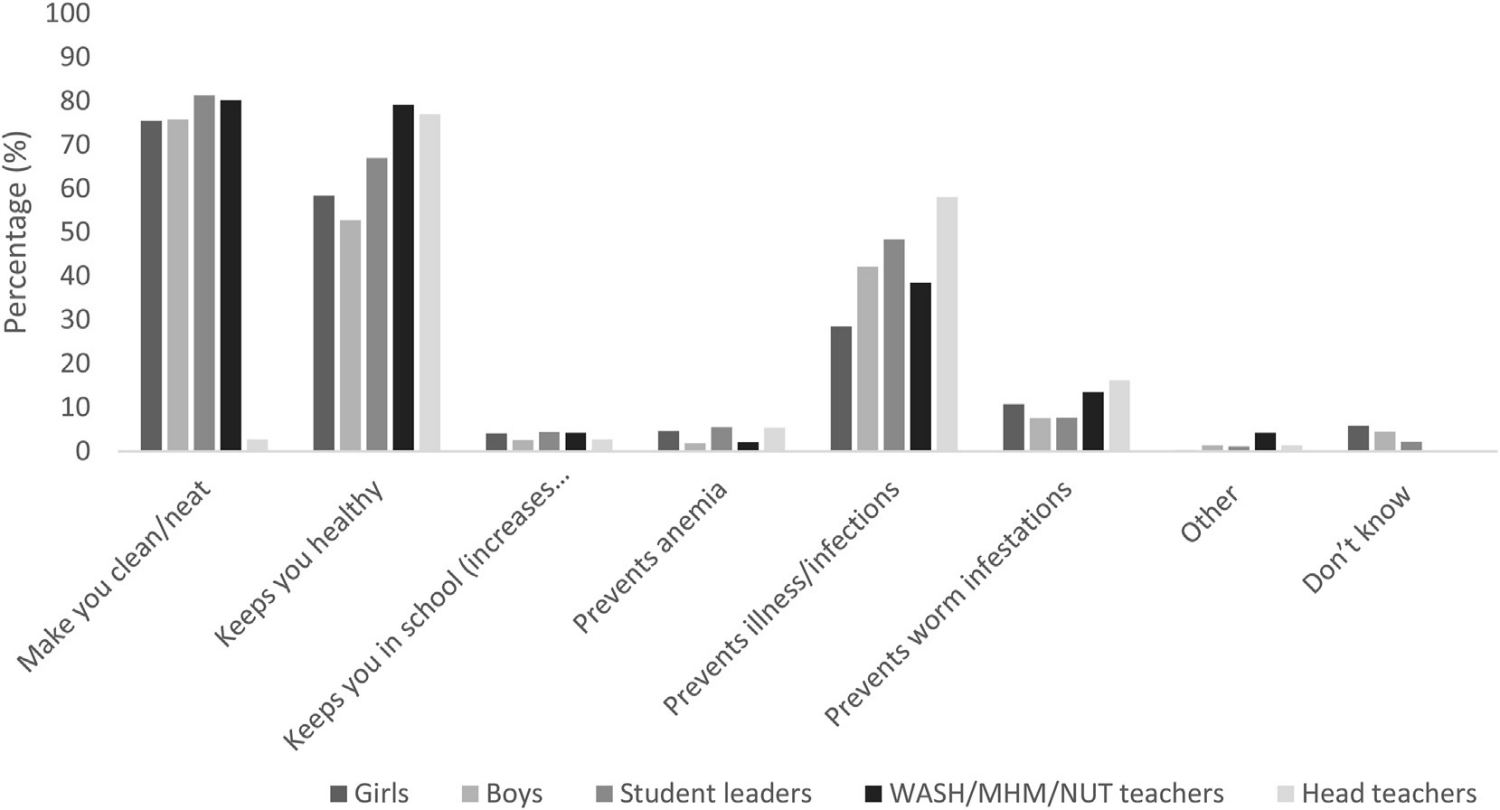
Reported benefits of keeping good Water, Sanitation and Hygiene (WASH) practices among adolescents and implementers at baseline for the School Nutrition for Adolescents Project (SNAP), Joyphurat district, Bangladesh, 2019. Girls: *N* = 2244; boys *N* = 773; student leaders *N* = 91; WASH/MHM/NUT teachers: *N* = 96; and head teachers: *N* = 74. MHM, menstrual hygiene management; NUT, nutrition-WIFA; WASH, water, sanitation, and hygiene.

Around 70%–80% of students and WASH/MHM/Nutrition-WIFA teachers stated that the benefit of good WASH practices is being clean and neat. The majority of adolescents and teachers reported that good WASH practices keep one healthy (range: 53%–79%). Around ≥40% adolescent boys and teachers stated that it prevents illness and infections, and among adolescent girls, only 29% reported this benefit (Figure 3).

### Attitudes and practices of adolescent girls and boys on school WASH

As shown in Table 2, almost all adolescent girls and boys reported that there was drinking water available at school and said they drank the water at school. When asked if they drank water provided by school on the day prior to the interview, 93% and 78% of girls and boys, respectively, responded yes, with virtually all reporting no challenges to drinking the water provided by the school.

**TABLE 2:** Adolescent girls’ and boys’ experiences with water, sanitation, and hygiene at school at baseline for the School Nutrition for Adolescents Project (SNAP) in Joypurhat, Bangladesh, 2019

	Adolescent girls N = 2244 n (%)	Adolescent boys N = 773 n (%)
Report water available at school and drink water at school	2184 (97.3)	727 (94.0)
Drank water provided by school previous day	**N = 2184**	**N = 727**
Yes	2023 (92.6)	568 (78.1)
Reported no challenges to drinking water at school	2112 (96.7)	706 (97.1)
Wash hands with soap before eating at school	**N = 2244**	**N = 773**
All the time or most of the time	261 (11.6)	94 (12.2)
Sometimes	825 (36.8)	151 (19.5)
Do not eat at school	273 (12.2)	188 (24.3)
Never	885 (39.4)	340 (44.0)
Wash hands with soap and water after using the toilet in school		
All the time	311 (13.9)	193 (25.0)
Sometimes	366 (16.3)	114 (14.7)
Rarely	590 (26.3)	85 (11.0)
Never	971 (43.3)	370 (47.9)
Ever use toilet at school	2011 (89.6)	593 (76.7)
Used toilet at school yesterday or today	**N = 2011**	**N = 593**
Yes	1257 (62.5)	302 (50.9)
Ever felt the need to use the toilet but purposely waited until could use a different toilet outside of school	**N = 2244**	**N = 773**
All the time	69 (3.1)	32 (5.4)
Sometimes	873 (38.9)	106 (17.9)
Never	1302 (58.0)	455 (76.7)
Ever avoid drinking or eating so that you do not have to use the toilet when you are at school		
No	2160 (96.3)	755 (97.7)
n’s vary due to missing data		

Around 4 out of 10 girls and boys said that they never washed their hands with soap and water before eating at school, or washed their hands with soap and water after using the toilet in school (43% girls, 48% boys). The majority (69% girls, 59% boys) reported that the lack of soap was a challenge to washing hands with soap and water at school (data not shown).

About 90% of girls and 77% of boys had ever used the toilet at school, out of which 63% and 51%, respectively, used it the day before or on the day of the survey. About 42% of girls and 23% of boys reported that all the time or sometimes, they felt the need to use the toilet but purposely waited until they could use a different toilet outside their school. Among girls and boys who always or sometimes waited to use a different toilet outside school, the most common reasons were that the toilets in the school were either over-crowded, not clean, or had no soap (data not shown). About 1% of girls and boys reported that they avoided drinking or eating so that they did not have to use the toilet when they were at school.

### Experiences of adolescent menstruating girls with menstrual hygiene management

As shown in Table 3, among the girls that had started menstruation (89%), 12.3 y was the average reported age when menstruation began. The preferred menstrual product reported by menstruating adolescent girls was disposable sanitary towels (80%) followed by reusable or washable cloths (20%), and 81% reported using sanitary towels when menstruating. Among girls who reported that they use sanitary towels when menstruating, 11% reported that getting sanitary towels was hard and procurement was mostly done by themselves in a store (82%). Among those not using sanitary towels, nearly all reported using reusable/washable cloth when menstruating (data not shown). Two-thirds of menstruating girls reported always attending school when menstruating, and a third of girls reported missing school sometimes when menstruating. Among girls who sometimes missed or did not attend school during menstruation, illness (73%) and discomfort (25%) were the main reasons reported. However, among all menstruating girls, 39% reported never having to leave school early due to the onset of unexpected menstruation at school. Never getting sanitary towels at school was uncommon (6%); among these girls (*n* = 130), they reported that they had got sanitary products at school on an average of 1–2 times in the past 3 mo (data not shown). Among the same girls that ever received a sanitary towel at school, in the past 3 mo, the mean number of times they received (free or purchased) was 1.4 times (data not shown). Almost 9 out of 10 menstruating girls indicated that there was not an adult at school that provided sanitary towels for sale or free.

**TABLE 3:** Adolescent menstruating girls’ experiences with menstrual hygiene management, at baseline for the School Nutrition for Adolescents Project (SNAP) in Joypurhat, Bangladesh, 2019

	Adolescent menstruating girls N = 2172 n (mean or %)
Mean age of menarche, y	2172 (12.3)
Preferred menstrual product	
Sanitary towels	1727 (79.8)
Reusable /washable cloth	437 (20.3)
Other	8 (0.4)
Uses disposable sanitary towels when menstruating	1765 (81.3)
Among those that use disposable sanitary towels when menstruating, how easy it is to get sanitary towels	**N = 1765**
Very easy or somewhat easy	1482 (84.0)
Not easy or hard	81 (4.5)
Very hard or somewhat hard	202 (11.4)
Among those that use disposable sanitary towels when menstruating, sanitary towel procurement^1^	
Store, girl buys them	1443 (82.0)
Store, household member buys them	265 (15.1)
Other (including school)^2^	51 (2.9)
Attends school when menstruating	**N = 2172**
Always	1399 (62.3)
Sometimes	675 (30.1)
Never	98 (4.4)
Among those who miss school when menstruating sometimes or never, why they miss	**N = 773**
Discomfort	191 (24.7)
Illness	564 (73.0)
Other	16 (2.3)
Ever left school early because of unexpected menstruation	**N = 2172**
Yes	850 (39.2)
Adult at school provides disposable sanitary towels	
Yes, for sale	105 (4.8)
Yes, for free	125 (5.8)
No	1927 (88.7)
Ever got sanitary products at school	130 (6.0)

N’s vary due to missing data.

1 Multiple responses could be reported.

2 Seven respondents indicated “at school” as a place that they received sanitary towels.

### Micronutrient status of adolescent girls by select characteristics

At baseline, the mean (SD) hemoglobin concentration, median (Q_1_, Q_3_) RBP concentration, serum ferritin concentration, RBC folate concentration, and serum folate concentration among adolescent girls aged 10–19 y were 12.6 (1.3) g/dL, 1.1 (0.9, 1.3) μmol/L, 44.1 (29.2, 62.8) μg/L, 527.5 (400.2, 732.1) nmol/L, and 12.1 (9.2, 16.2) nmol/L, respectively (data not shown).

Table 4 shows the prevalence of anemia, ID, IDA, vitamin A deficiency, risk of serum folate deficiency, and RBC folate insufficiency by characteristics among adolescent girls. One in 4 adolescent girls had anemia, which varied by ethnicity ranging from 24% among Bengalis to 61% among other ethnic groups. ID was 9% but varied by age group, household socioeconomic status (SES), and menstruation status, with almost double the prevalence of ID among low-income than high-income households and a prevalence of only 1.5% among those who had not reached menarche. IDA (4%) and vitamin A deficiency (3%) were low. The risk of serum folate deficiency was 10% but varied by age group and menstruation, with younger girls and those not yet reaching menarche having lower prevalence (8.7% and 1.5%, respectively). RBC folate insufficiency was common (76%) and differed by age group, ethnicity, and household SES, with 100% of the other ethnic groups having insufficiency and the high SES group having higher RBC folate insufficiency compared to the low SES group. The prevalence of RBC folate deficiency (total = 3%) and the risk of RBC folate deficiency (total = 9%) among adolescent girls varied by ethnicity and household SES, with folate deficiency and the risk of folate deficiency being highest (5.3% and 14.8%, respectively) in the high SES group (data not shown).

**TABLE 4:** Prevalence of anemia, ID, IDA, vitamin A deficiency, risk of serum folate deficiency, and RBC folate insufficiency by characteristics among adolescent girls aged 10–19 y at baseline for the School Nutrition for Adolescents Project (SNAP), Joyphurat district, Bangladesh, in 2019

Characteristics		Anemia^1^ N = 2150	ID^2^ N = 2038	IDA^3^ N = 2038	Vitamin A deficiency^4^ N = 2038	Risk of serum folate deficiency^5^ N = 2159	RBC folate insufficiency^6^ N = 2159
		n (%)					
Age, y	10-13.9	331 (23.5)	108 (8.1) ^∗∗^	46 (3.4)	44 (3.3)	123 (8.7) ^∗∗^	1045 (73.9) ^∗∗^
	14-18.9	201 (27.1)	78 (11.2)	33 (4.7)	23 (3.3)	86 (11.6)	599 (80.5)
Ethnicity	Bengali	513 (24.2) ^∗∗^	180 (8.9)	76 (3.8)	65 (3.2)	204 (9.6)	1613 (75.8) ^∗∗^
	Other^7^	19 (61.3)	6 (20.7)	3 (10.3)	2 (6.9)	5 (16.1)	31 (100.0)
Socioeconomic status	Low	210 (24.6)	93 (11.6) ^∗∗^	43 (5.3) ^∗∗^	25 (3.1)	80 (9.3)	608 (71.0) ^∗∗^
	Middle	212 (24.5)	67 (8.1)	25 (3.0)	22 (2.7)	91 (10.5)	683 (78.5)
	High	110 (25.5)	26 (6.3)	11 (2.7)	20 (4.9)	38 (8.8)	353 (81.5)
Menarche	Yes	515 (24.7)	185 (9.4) ^∗∗^	79 (4.0)	64 (3.2)	208 (9.9) ^∗∗^	1598 (76.4)
	No	16 (24.2)	1 (1.5)	0 (-)	3 (4.6)	1 (1.5)	44 (66.7)
Total		**532** (**24.7)**	**186** (**9.1)**	**79** (**3.9)**	**67** (**3.3)**	**209 (9.7)**	**1644 (76.1)**

*P* values test for differences between groups at baseline by Rao–Scott chi-square or Fisher’s exact test for the overall population for categorical variables. All estimates accounted for clustering. N’s are unweighted.

1 Anemia – children aged 10–11 y: hemoglobin (Hb) < 11.5 g/dL; girls ≥ 12 y: Hb < 12 g/dL [26]. Hb values unadjusted for altitude or smoking because the Joypurhat study area is not above 1000 m and smoking is rare among adolescent girls in Bangladesh.

2 ID: serum ferritin < 15 μg/L, adjusted for inflammation based on a–1 acid glycoprotein (AGP) and CRP based on BRINDA [27, 30].

3 IDA: serum ferritin < 15 μg/L + anemia, adjusted for inflammation based on AGP and CRP based on BRINDA [27, 30].

4 Vitamin A deficiency: RBP < 0.7 μmol/L [32].

5 Risk of serum folate deficiency: serum folate < 7 nmol/L; deficiency based on hematologic indicator [23].

6 RBC folate insufficiency: folate insufficiency in RBCs for preventing neural tube defects: RBC folate <748nmol/L using 5MeTHF assay-specific cutoff [23].

7 Other included Adibasi, Gonju and Maly, Chakma, Santhals, Garo, Urow, and Rajbonshi ethnic groups. ^∗∗^*P* value < 0.05 (significant difference between groups).

### School-level observations of WASH facilities

As shown in Table 5, around 70% of schools had drinking water from an improved source available at the time of the survey and met the Sustainable Development Goals (SDG) criteria for “basic” drinking water service. In the 70 schools where water samples were collected, more than 40% had at least one of the 2 drinking water samples contaminated with *E. coli* and were noncompliant with the WHO standards [34].

**TABLE 5:** Core∗ indicators for monitoring basic water, sanitation, and hygiene by observation in 74 schools at baseline for the School Nutrition for Adolescents Project (SNAP) in Joypurhat, Bangladesh, 2019

Project domains	Indicator/Measure	Proportion of schools, n (%) (N = 74)
Water	Proportion of schools with at least one main improved^1^ drinking water source	71 (95.9)
	Proportion of schools with at least one main drinking water available^2^ from an improved source	50 (70.4)
	Proportion of schools with all sampled drinking water sources compliant^3^ with WHO standard for E. coli	41 (58.6)^4^
Sanitation	Proportion of schools with at least one improved^5^ toilet	74 (100.0)
	Proportion of schools with at least one improved toilet that is useable^6^	36 (48.6)
	Proportion of schools with at least one improved toilet that is single sex	66 (89.2)
	Proportion of schools with at least one improved toilet that is usable and single sex	31 (41.9)
Hygiene	Proportion of schools with at least one handwashing facility^7^, which has water available	55 (78.6)^8^
	Proportion of schools with at least one handwashing facility which has both water and soap available^9^	2 (2.9)^8^

1 The main drinking water source is adequately protected from outside contamination and includes tube well, protected dug well or piped water.

2 Sustainable Development Goals indicator for basic drinking water service: the main source of drinking water is an improved source and drinking water is available from the main source at the school at the time of the survey.

3 Compliant- Absence of any *E. coli* in 100mL water sample based on WHO criteria.

4 Among the 70 schools that provided water samples.

5 Type of sanitation facility that are flush/pour-flush toilets, pit latrines with slab, or compositing toilets.

6 Sanitation facilities are available to students (doors are unlocked or a key is always available), functional (toilet is not broken, toilet hole is not blocked, water is available for flush/pour-flush toilets), and private (there are closable doors that lock from the inside and no large gaps in the structure) at the time of the survey.

7 Any device or infrastructure with running water that is available for handwashing at the time of the survey.

8 Among the 70 schools with at least one handwashing facility present.

9 Sustainable Development Goals indicator for basic hygiene service: defined as the presence of at least 1 handwashing facility providing running water and soap at the time of the survey.

∗ WASH standards and criteria were adapted from WHO/UNICEF, 2016. Core questions and indicators for monitoring WASH in Schools in the Sustainable Development Goals [25].

The observation checklist allowed for observing 6 facilities per school, so a maximum of 6 facilities per school were observed and there may have been more facilities. All schools had at least 1 improved toilet facility for student use; however, less than half (49%) had at least one facility that met the SDG definition for useability (available, functional, and private), and only about 42% had an improved toilet that was usable and single-sex and met the SDG criteria for “basic” sanitation service. There was a median of 72 girls per usable toilet/latrine and 84 boys per usable toilet/latrine in schools with single-sex toilets. In 14% of schools, at least one toilet/latrine was shared by students and teachers (data not shown).

Most schools (95%) had handwashing facilities present on the day of the survey; however, among these, only 79% of the facilities had water availability. Only 2 schools (3%) had both soap and water available and met the SDG criteria for “basic” hand hygiene service.

## Discussion

Findings from this baseline survey among adolescent girls, boys, and project implementers prior to starting a school-based integrated nutrition and health intervention project in rural Bangladesh show variable awareness, KAP, and coverage of nutrition and health topics and interventions within schools. Although relatively fewer adolescent girls and boys were exposed to nutrition and health topics including anemia, IFA tablet, deworming, and worm infestations, more project implementers (headteachers, teachers, and student leaders) were aware of these topics at baseline. The low nutrition and health knowledge, minimal baseline IFA supplement intake among adolescent girls, the high numbers reporting of ever having had a worm infestation, and the percentage of girls missing school during menstruation or ever leaving school early due to unexpected menstruation are indications that the BCI classroom sessions and provision of WIFA, WASH, and MHM supplies planned as part of the SNAP intervention project could address key gaps and potentially improve practices in this school health and nutrition context. Particularly with adolescent girls being expected to consume WIFA at school, the lack of safe water and soap to meet “basic” WASH services are critical issues that all projects considering similar interventions should take note of in order to ensure that all students have access to basic WASH services at school [25].

The SNAP intervention design was intended to support changing the KAP of nutrition-WIFA and WASH, including improving dietary diversity and quality; increase coverage and intake adherence to weekly IFA supplementation; improve WASH and MHM behaviors; and support availability of relevant WASH and MHM supplies within schools. Including adolescent boys in the nutrition-related BCI sessions for improving nutritional wellbeing was a priority to motivate boys to understand the nutritional requirements of themselves and other household members as the boys grow into adulthood. The expectation was that the combined effect of the intended changes would reduce anemia, other micronutrient deficiencies, morbidity, and infections, and improve the retention and attendance of adolescent menstruating girls in schools. In recent years, integrated health and nutrition intervention studies with similar designs such as SNAP have been shown to improve maternal and infant health and nutrition outcomes in LMICs [[35], [36], [37], [38], [39], [40]]. Nguyen et al. [35] found in rural Bangladesh that integrating nutrition-focused interpersonal counseling, community mobilization, distribution of free IFA and calcium supplements, and weight-gain monitoring through an existing Maternal, Neonatal, and Child Health (MNCH) program improved maternal dietary diversity, adherence to supplementation, and exclusive breastfeeding practices compared to the standard MNCH program. Similarly, a meta-analysis of 34 studies including 11 in LMICs showed that the impact of nutrition education and counseling greatly improved gestational weight gain and birth weight and reduced the risk of anemia in late pregnancy and preterm birth when combined with direct nutrition support, including food, multiple micronutrient supplements, and nutrition safety nets [36]. Similarly, integrated nutrition interventions among in-school and out-of-school adolescents have proved to be effective in reducing anemia and other nutrition and health-related problems. In India, IFA supplementation combined with nutrition education on micronutrient deficiencies and healthy eating both in and out of school improved adherence to supplementation and reduced anemia in adolescent girls [41].

We found that 1 in 4 adolescent girls had anemia, similar to the reported 26% anemia prevalence in NPNL (including adolescents) during the Bangladesh National Micronutrient Survey (NMS) 2011–2012 [1] and the global prevalence of anemia (2000-19) [7], but lower than the reported 40% prevalence in the general population during the Bangladesh Demographic and Health Survey conducted in the same year as the NMS 2011-12, which measured Hb using a single drop of capillary blood and a HemoCue 201 [42]. In our survey, we used venous blood to assess Hb using the HemoCue 301, and it is known that Hb concentrations are influenced by sampling techniques and that venous is the preferred blood source over a capillary drop sample [[43], [44], [45], [46]]. The persistence of anemia as a moderate public health problem (20<40% as defined by WHO) [47], among NPNL women particularly in rural Bangladesh for over a decade, reinforces the fact that undernutrition among vulnerable groups in the country is still a concern. This warrants reviewing of the existing public health programs, strategies, and platforms to reach adolescents, particularly the school-level platform because for girls who attend schools consistently, the schools are opportunistic to support sustained high coverage and use of interventions. Reports from the National Strategy on the Prevention and Control of Micronutrient Deficiencies in Bangladesh (2015–2024) confirm that current routine nationwide efforts that provide interventions to prevent and reduce anemia do not focus much on adolescent girls and identified several contributory factors including the lack of political commitment, irregular IFA tablet supply, public misconceptions about IFA intake, and poor coverage of vulnerable groups in hard-to-reach areas [48]. National anemia prevention and control programs may need to address these problems to inform policy and reduce anemia. Anemia prevalence varied by ethnicity in our sample, which could be attributed to differences in dietary preferences, cultural, and social practices among such groups as reported in similar studies in Bangladesh [49], India [50], and worldwide [51].

The prevalence of ID was 9% among adolescent girls in our sample and was significantly lower among younger (10–14 y) adolescent girls, girls from high household SES, and nonmenstruating girls. The prevalence of ID in NPNL women during the NMS 2011–2012 was 7% [1], comparable to the prevalence that we found in our sample. Similar studies in Bangladesh [19, 52, 53] reaffirm the low prevalence of ID in some parts of the country, which has generally been attributed to high concentrations of iron in groundwater primarily consumed in households [19, 54]. This is in line with findings from our survey where about a 5th of adolescents reported that they thought there was iron in their household water, although <10% thought the drinking water tasted or smelled like rust or had a red or rusty color. In our sample, over 60% of girls had a minimum dietary diversity score of >5, although only about 40% consumed rich sources of heme iron. This suggests that although the diets of adolescents in this population may be diverse, consumption of iron in the form of iron-rich animal source foods may be lacking. IDA was quite low (4%) in our sample, similar to the national prevalence of 5% among NPNL women in the NMS 2011–2012 [1]. Although ID has been considered to be the largest cause of anemia globally, other underlying factors could be play a more significant role in the etiology of anemia in this sample.

Folate deficiency, about 10% in our sample, has been associated with anemia among women in Bangladesh [55] and might be playing a more defined role in the prevalence of anemia in this population. In this sample, the risk of serum folate deficiency was highest among older girls (15–19 y), whereas RBC folate insufficiency, deficiency, and risk of deficiency were all highest among girls from high household SES. It is uncertain why the prevalence of folate deficiency was highest among older or girls from wealthier families, as undernutrition would potentially be assumed to be higher with poor wealth status; however, girls from wealthier households in our sample consumed less diversified diets than those from poorer households, which may have been a contributory factor. Folate is important for preventing anemia and neural tube defects during pregnancy and other negative birth outcomes [10] and 3 out of 4 of the girls in this survey had insufficiency; thus, national efforts for prevention and treatment of its insufficiency and deficiency may be needed.

Vitamin A deficiency in our sample was low (3%), although only about 1 out of 4 and 1 out of 5 girls reported that they consumed vitamin A–rich fruits or vegetables, respectively. The low prevalence may be due to the existing programs in the country to prevent vitamin A deficiency, including the fortification of edible oils with vitamin A and rice fortification with vitamin A and other micronutrients instituted by the GoB in the past decade [1].

In our sample, experiences of adolescent girls on MHM in school leaves room for improvement. About a third of the girls reported that they sometimes missed school during menstruation and a quarter of the girls said they never had to leave school due to unexpected menstruation. In the most recent Bangladesh National Hygiene Survey (BNHS) in 2018 [56], a third of schoolgirls reported missing an average of 2.5 d a month due to menstruation, which is comparable to the findings from our survey among schoolgirls in the Joypurhat district. This suggests an important need for in-school facilities to provide infrastructure for the supply and disposal of MHM materials, including the provision of disposable sanitary pads, sex-appropriate toilets that are equipped to accommodate MHM needs, operations and maintenance of WASH facilities, and education and guidance for girls on menstruation to improve girls’ school attendance and retention. Ongoing efforts by UNICEF, the GoB, and nongovernmental organizations to integrate MHM into WASH in schools, adolescent health, nutrition, and education sector programs and activities to reduce the price and waive taxes on disposable sanitary pads and products [57,58] may support these efforts and girls’ school attendance.

School water consumption was high among adolescents, which could potentially cause illness including urinary tract and intestinal infections since 40% of schools’ sampled drinking water access points were contaminated with *E. coli* (an indication of fecal matter in the water supply [59]). Additionally, observations of facilities within schools showed substandard basic hygiene services, with nearly nonexistent soap availability at handwashing facilities for student use. However, it should be recognized that observation checklists only allowed for observations of 6 facilities maximum per school and there may have been >6 facilities in a school. By observation, less than 50% of the schools met the SDG criteria for basic sanitation services; responses from students indicated the lack of cleanliness and soap and overcrowding of toilet facilities in schools as common reasons to why they avoided using toilet. In Bangladesh, the WHO recommended toilet to schoolgirl ratio is 1:25 [58]. In our sample, this was about 3 times higher (1:72), but twice better than the 1:179 ratio reported in a similar baseline survey in the Netrokona district of Bangladesh in 2017 [60], an indication of poor sanitation services within surveyed schools. Results of a randomized controlled trial conducted in Kenya showed that the presence of an appropriate WASH environment, including hygiene promotion, water treatment, and access to sanitation, increased schoolgirls’ attendance by almost 60% [61]. In studies from Nepal, Pakistan, and Bangladesh [62, 63], poor toilet and water facilities were associated with an increased risk of anemia, making WASH behavior at the individual and school level a great concern to be addressed. More so, for IFA programs to be run effectively in the school setting, adequate and safe drinking water supply is important to ensure adherence and avoid infection with waterborne diseases when consuming IFA.

Reducing and/or preventing anemia or ID in this population may go beyond the recommended single intervention efforts by the WHO and Bangladesh IPHN in the form of WIFA supplementation for NPNL women and adolescent girls [64,65] to encompass an integrated approach of nutrition and WASH (and MHM) interventions at the school and community level. Furthermore, since iron and vitamin A deficiency are low in this population, there is room for the GoB and other stakeholders to introduce other strategies for preventing and controlling micronutrient deficiencies, particularly multiple micronutrient supplements (MMS) for NPNL women including adolescent girls, although more evidence may be needed to inform acceptability, feasibility sustainability, equity, and cost-effectiveness [66], particularly for the adolescent age group. Evidence from effectiveness and cost-effectiveness studies in Bangladesh and Burkina Faso suggest that a policy change from the use of IFA supplements as part of antenatal care for pregnant women to the use of MMS would cost-effectively save lives by averting between 5000 to >15,000 deaths and 5000–30,000 cases of preterm births annually given 100% program coverage and adherence to supplementation [67]; other studies in Bangladesh found similar evidence of improved cost-effectiveness, including averting 1,268,067 disability adjusted life years (DALYs) and the value of DALYs averted estimated at $3,696,039,235 [68]. Additional benefits of pre- and post-natal MMS on maternal micronutrient status (besides iron and folate) and on child growth have also been shown in-country [69,70]. Thus, future considerations to strengthen maternal nutrition services in Bangladesh using prenatal MMS could also be tailored to the adolescent population.

A limitation of this study is the lack of data on girls’ anthropometry to determine their nutritional status or BMI categorization, which is a useful indicator to characterize the health status. Furthermore, we did not collect direct data of iron concentration in groundwater tube wells or dug wells, and thus, the association of perceived groundwater iron with the iron status of girls in our sample warrants cautious interpretation. Additionally, this instrument was validated only among adult women living in rural areas and not adolescents. Our self-reported measures for the survey, including the intake of IFA, deworming tablet, and 24-h dietary food group recall, and experiences with school WASH, MHM, and nutrition interventions or topics may have had a social desirability or recall bias that is also a limitation. WASH observations of facilities were limited to 6, and some schools may have had more facilities. This study has several strengths, including a robust study design using a representative sample of adolescent students in the Joypurhat district (excluding the Joypurhat Sadar upazila) and adds to the dearth of adolescent nutrition data in Bangladesh and globally. Only one of the 75 invited schools refused to participate, and thus, low attrition rates afforded higher statistical power for observed associations. We also collected data on several objective measures such as biomarkers for iron, vitamin A, and folate status, school water source testing for *E. coli*, and direct observations of WASH facilities.

In summary, this study highlights that 3 of 4 girls had folate insufficiency and 1 of 4 girls had anemia with varying levels of iron, folate, and vitamin A deficiencies in rural Bangladesh prior to the start of a school nutrition project. Over 60% of the adolescent girls and boys achieved minimum dietary diversity, although consumption of rich sources of heme iron is not optimal and there is variable awareness and coverage of nutrition interventions among adolescents and project implementers. School achievement of the WHO SDG basic sanitation and hygiene services are low for some indicators and *E coli* contamination of drinking water sources is present in about 40% of schools. The findings of this study suggest further review and consideration of a multipronged approach of integrated nutrition, MHM, and WASH interventions at the school level to improve adolescent nutrition and health.

## Funding

This study was supported by funding to the CDC Foundation, Atlanta, USA, by Nutrition International through its Institutional Support Grant and Nutrition Leverage and Influence for Transformation grants and by Global Affairs, Canada. We are extremely grateful for the information, guidance, and support provided by all; the authors alone are responsible for the content of this paper. The findings and conclusions in this manuscript are those of the authors and do not necessarily represent the official position of the Centers for Disease Control and Prevention.

## Author disclosures

The authors report no conflicts of interest. No one involved in the production of the manuscript received any honorarium, grant, or other form of payment.

## Data Availability

The data described in the manuscript, code book, and analytic code will not be made available because other publications are in progress, which will use the same dataset. Furthermore, the funding source’s current policy only allows datasets to be used by research partners.
