# Supplementary material for: Design, Methods, and Select Baseline Results from a School Nutrition Project for Adolescents in Bangladesh
Source: Curr Dev Nutr. 2023 Mar 30;7(4):100070. doi: 10.1016/j.cdnut.2023.100070 (PMC10257226; doi:10.1016/j.cdnut.2023.100070)
Supplement: Multimedia component 1 [file mmc1.docx]

Online Supporting Material: Design, Methods, and Select Baseline Results from a School Nutrition Project for Adolescents in Bangladesh

**SUPPLEMENTAL TABLES**

**Supplemental Table S1.** Adolescent Girls’ and Boys’ Age by Intervention Arm at Baseline for the School

Nutrition Adolescent Program (SNAP), Joyphurat district, Bangladesh, 2019

|  | **Full package** | | **Limited package** | | **Control** | | **Overall (Full + Limited + control)** | |
| --- | --- | --- | --- | --- | --- | --- | --- | --- |
|  | **n** | **Mean** | **n** | **Mean** | **n** | **Mean** | **n** | **Mean** |
| **Adolescent Girls** | **N=745** | | **N=788** | | **N=711** | | **N=2244** | |
| **Mean age (years)** | 745 | 14.1 | 788 | 14.1 | 711 | 14.1 | 2244 | 14.1 |
| **Adolescent Boys** | **N=254** | | **N=267** | | **N=252** | | **N=773** | |
| **Mean age (years)** | 254 | 14.3 | 267 | 14.3 | 252 | 14.3 | 773 | 14.3 |

**Supplemental Table S2.** Sex and age of Implementers by Intervention Arm for the School Nutrition for Adolescents Project (SNAP), Joyphurat district, Bangladesh, 2019

Not applicable, -. There were no intervention specific student leaders or teachers interviewed in Control schools

|  | **Full package** | | **Limited package** | | **Control** | | **Overall (Full + Limited + control)** | |
| --- | --- | --- | --- | --- | --- | --- | --- | --- |
|  | **n** | **%** | **n** | **%** | **n** | **%** | **n** | **%** |
|  | | | | | | | | |
| **Gender** | **N=** **25** | | **N=24** | | **N=25** | | **N=74** | |
| Male | 25 | 100.0 | 23 | 95.8 | 25 | 100.0 | 73 | 98.6 |
| Female | 0 | - | 1 | 4.2 | 0 | - | 1 | 1.4 |
| **Mean age, y** | **n** | **Mean** | **n** | **Mean** | **n** | **Mean** | **n** | **Mean** |
|  | 25 | 49.2 | 24 | 49.4 | 25 | 49.3 | 74 | 49.3 |
| **Student leaders** | | | | | | | | |
| **Gender** | **N= 66** | | **N=25** | | **N=0** | | **N=91** | |
| Male | 26 | 39.4 | 11 | 44.0 | - | - | 37 | 40.7 |
| Female | 40 | 60.6 | 14 | 56.0 | - | - | 54 | 59.3 |
| **Mean age, y** | **n** | **Mean** | **n** | **Mean** | **n** | **Mean** | **n** | **Mean** |
|  | 66 | 14.2 | 25 | 14.2 | - | - | 91 | 14.2 |
| **All teachers** | | | | | | | | |
| **Gender** | **N=** 74 | | **N=22** | | **N=0** | | **N=96** | |
| Male | 33 | 44.6 | 10 | 45.5 | - | - | 43 | 44.8 |
| Female | 41 | 55.4 | 12 | 54.5 | - | - | 53 | 55.2 |
| **Mean age, y** | **n** | **Mean** | **n** | **Mean** | **n** | **Mean** | **n** | **Mean** |
|  | 74 | 42.1 | 22 | 39.3 | - | - | 96 | 41.4 |

Legends for Supplemental Figures

**SUPPLEMENTAL FIGURE S1**

Study profile showing adolescent girls and boys who were enrolled into the baseline survey for the School Nutrition for Adolescents Project (SNAP), Joyphurat district, Bangladesh, 2019, and the reasons some were lost to follow-up. Full Package (FP) intervention, included all components of the project (Weekly Iron and Folic Acid (WIFA) supplementation, Menstrual Hygiene Management (MHM), Water, Sanitation and Hygiene (WASH), and Behavior Change Intervention (BCI)). Limited Package (LP) intervention, included only WIFA supplementation and BCI components. Control group, adolescent girls and boys did not receive any intervention component.

**SUPPLEMENTAL FIGURE 2**

Response rates of implementers for the School Nutrition for Adolescents Project (SNAP), Joyphurat district, Bangladesh, 2019. Limited Package (LP) intervention included only WIFA supplementation and BCI components. Control group, adolescent girls and boys did not receive any intervention component.
