# Supplementary material for: Design, Methods, and Select Baseline Results from a School Nutrition Project for Adolescents in Bangladesh
Source: Curr Dev Nutr. 2023 Mar 30;7(4):100070. doi: 10.1016/j.cdnut.2023.100070 (PMC10257226; doi:10.1016/j.cdnut.2023.100070)

Demuyakor, M.E Design, Methods, and Select Baseline Results from a School Nutrition Project for Adolescents in Bangladesh

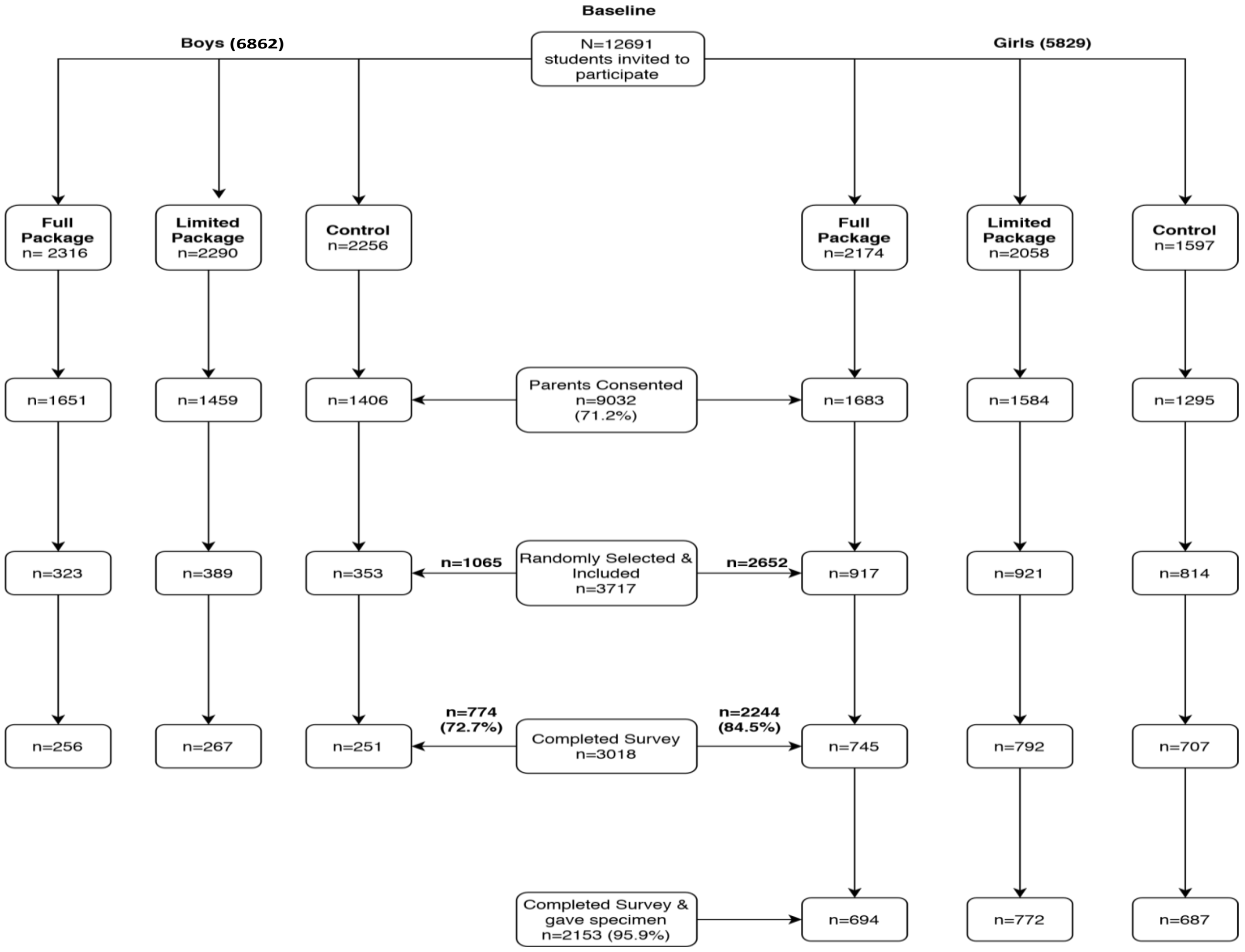

Supplement: Multimedia component 2 [file mmc2.pdf]
